# Supplementary material for: Evaluation of the Hematological Patterns from Up to 985 Days of Long COVID: A Cross-Sectional Study
Source: Viruses. 2023 Mar 29;15(4):879. doi: 10.3390/v15040879 (PMC10142608; doi:10.3390/v15040879)
Supplement: Supplementary file 1 [file viruses-15-00879-s001.zip › viruses-2274879-supplementary.pdf]

Table S1. Adopted reference ranges for laboratory exams.

Exam	RR
RBCs, millions/mm^3	4 – 5,6
Haemoglobin, g/dL	12 – 16,5
Haematocrit, %	35 – 47
MCV, fL	81 – 101
MCH, pg	27 – 34
MCHC, %	31,5 – 36
RDW, %	11,5 – 16
ESR, mm	<15[(a)], <20[(b)], <30[(c)]
WBCs, thousands/mm^3	5 - 10
Neutrophils thousands/mm^3	3 – 5
Eosinophils, /mm^3	100 – 300
Basophils, /mm^3	0 – 100
Monocytes, /mm^3	200 – 400
Lymphocytes, thousands/mm^3	1,5 – 2,5
Platelets, thousands/mm^3	150 – 450
MPV, fL	6,2 – 11
Plateletcrit, %	0,150 – 0,425
PDW, %	18 – 22
PT, s	11 – 15
PT activity, %	70 – 180
aPTT, s	24 – 39

(a), males <50 years; (b), males ≥50 years / females <50 years; (c), females ≥50 years. RR, reference range; RBCs, red blood cells; MCV, mean corpuscular volume; MCH, mean corpuscular haemoglobin; MCHC, mean corpuscular haemoglobin concentration; RDW, red cell distribution width; ESR, erythrocyte sedimentation rate; WBCs, white blood cells; MPV, mean platelet volume; PDW, platelet distribution width; PT, prothrombin time; aPTT, activated partial thromboplastin time.
